# Suppression of the hERG potassium channel response to premature stimulation by reduction in extracellular potassium concentration

**DOI:** 10.14814/phy2.12165

**Published:** 2014-10-15

**Authors:** Dario Melgari, Chunyun Du, Aziza El Harchi, Yihong Zhang, Jules C. Hancox

**Affiliations:** 1 School of Physiology and Pharmacology and Cardiovascular Research Laboratories, University of Bristol, Medical Sciences Building, Bristol, BS8 1TD, UK

**Keywords:** *Human ether‐à‐go‐go‐related gene*, hyperkalemia, hypokalemia, long QT, potassium, potassium channels, QT interval

## Abstract

Potassium channels encoded by *human ether‐à‐go‐go‐related gene* (*hERG*) mediate the cardiac rapid delayed rectifier K^+^ current (*I*_Kr_), which participates in ventricular repolarization and has a protective role against unwanted premature stimuli late in repolarization and early in diastole. Ionic current carried by hERG channels (*I*_hERG_) is known to exhibit a paradoxical dependence on external potassium concentration ([K^+^]_e_), but effects of acute [K^+^]_e_ changes on the response of *I*_hERG_ to premature stimulation have not been characterized. Whole‐cell patch‐clamp measurements of hERG current were made at 37°C from hERG channels expressed in HEK293 cells. Under conventional voltage‐clamp, both wild‐type (WT) and S624A pore‐mutant *I*_hERG_ during depolarization to +20 mV and subsequent repolarization to −40 mV were decreased when superfusate [K^+^]_e_ was decreased from 4 to 1 mmol/L. When [K^+^]_e_ was increased from 4 to 10 mmol/L, pulse current was increased and tail *I*_hERG_ was decreased. Increasing [K^+^]_e_ produced a +10 mV shift in voltage‐dependent inactivation of WT *I*_hERG_ and slowed inactivation time course, while lowering [K^+^]_e_ from 4 to 1 mmol/L produced little change in inactivation voltage dependence, but accelerated inactivation time course. Under action potential (AP) voltage‐clamp, lowering [K^+^]_e_ reduced the amplitude of *I*_hERG_ during the AP and suppressed the maximal *I*_hERG_ response to premature stimuli. Raising [K^+^]_e_ increased *I*_hERG_ early during the AP and augmented the *I*_hERG_ response to premature stimuli. Our results are suggestive that during hypokalemia not only is the contribution of *I*_Kr_ to ventricular repolarization reduced but its ability to protect against unwanted premature stimuli also becomes impaired.

## Abstract (precis)

hERG potassium channels are important for ventricular repolarization and for protecting the ventricles of the heart from unwanted premature stimuli. This study shows that, in addition to reducing the contribution of hERG channel current to ventricular repolarization, hypokalemia impairs the protective response of hERG to premature stimulation.

## Introduction

Repolarization of cardiac action potentials (APs) depends on the interplay between inward and outward conductances during the AP plateau, with key roles identified for several potassium ion channels (Tamargo et al. 2004). *hERG (human ether‐à‐go‐go‐related gene)* encodes a protein that underlies the pore‐forming subunit of potassium channels mediating the rapid delayed rectifier current, *I*_Kr_ (Sanguinetti et al. 1995; Trudeau et al. 1995). Due to fast voltage‐dependent inactivation, *I*_Kr_/hERG channels pass little current at the peak of the ventricular action potential (AP), but mediate greater current as the AP plateau proceeds, peaking before the final rapid repolarization phase of the AP (Hancox et al. 1998; Zhou et al. 1998), which is mediated by a different potassium current (the inward rectifier, *I*_K1_; Shimoni et al. 1992; Mitcheson and Hancox 1999). Loss‐of‐function mutations in *hERG* are associated with the LQT2 form of the Long QT Syndrome (LQTS; Modell and Lehmann 2006), while gain‐of‐function *hERG* mutations are associated with the SQT1 variant of the short QT syndrome (SQTS; Brugada et al. 2004; Sun et al. 2011).

When hERG was initially identified, the magnitude of hERG current (*I*_hERG_) was demonstrated to have an anomalous dependence on extracellular K^+^ concentration ([K^+^]_e_), with low‐[K^+^]_e_ reducing outward *I*_hERG_ amplitude and raised [K^+^]_e_ augmenting the current (Sanguinetti et al. 1995). These changes were the opposite of those expected due merely to changes in electrochemical gradient and were observed also for native *I*_Kr_ (Sanguinetti and Jurkiewicz 1992; Yang and Roden 1996). This anomalous [K^+^]_e_ dependence of *I*_Kr_ was subsequently proposed to arise from the rectification properties of the *I*_Kr_ channel and specifically that rapid inactivation underlies this effect (Yang et al. 1997), most likely because external K^+^ ions interact with the pore and influence the channel's rapid collapse‐of‐pore type inactivation (Smith et al. 1996). This property of *I*_Kr_/hERG has clinical significance as, on the one hand, hypokalemia can exacerbate effects of QT interval prolonging, hERG‐blocking drugs (Hancox et al. 2008) whilst, on the other hand, potassium supplementation has been reported to improve repolarization in some LQT2 patients (Compton et al. 1996; Etheridge et al. 2003).

In addition to their role in ventricular AP repolarization, due to comparatively slow deactivation kinetics, *I*_Kr_/hERG channels can also contribute to net membrane conductance early in diastole and may play a protective role against premature beats (Smith et al. 1996; Lu et al. 2001). Consistent with this, using the “AP clamp” technique, Lu et al. (2001) demonstrated that premature stimuli applied late during AP repolarization or early in diastole elicit rapid outward *I*_hERG_ transients that would be anticipated to oppose premature depolarization. Subsequent studies have demonstrated that this property can be altered by LQTS gene mutation (Lu et al. 2003) or acidosis (Du et al. 2010). As both the magnitude and inactivation properties of *I*_Kr_/hERG are considered sensitive to [K^+^]_e_, a question of significance is whether or not the putative protective role of hERG against premature stimulation is altered by [K^+^]_e_? Accordingly, the aim of this study was to address this question through a combination of conventional and AP voltage‐clamp experiments on recombinant hERG channels.

## Methods

### Wild‐type and S624A hERG channels constructs

Human Embryonic Kidney (HEK‐293) cells stably expressing wild‐type (WT) hERG channels construct were donated by Prof Craig January (Zhou et al. 1998). The S624A mutant was generated using QuickChange^®^ (Stratagene, La Jolla, CA) mutagenesis as described previously (El Harchi et al. 2012). hERG 1b in pcDNA3.1 was donated by Prof Gail Robertson.

### Cells maintenance and transfection

HEK‐293 cells stably expressing WT hERG or transiently expressing S624A‐mutant constructs were maintained and passaged as described previously (Zhang et al. 2011; El Harchi et al. 2012). Cells were transfected 24–48 h after plating in 40 mm petri dishes. Transient transfections were conducted using Lipofectamine™ LTX (Life Technologies, Carlsbad, CA) following the instructions provided by the manufacturer. To mark successful transfections, 0.5 *μ*g of S624A‐mutant construct were always cotransfected with 1.0 *μ*g of green fluorescent protein (GFP, in pCMX donated by Dr. Jeremy Tavare, University of Bristol, UK). For experiments on coexpressed hERG1a/1b, 0.25 *μ*g of the hERG 1a construct were cotransfected with the same amount of hERG 1b, together with 0.5 *μ*g of CD8 as a transfection marker. Successfully transfected cells were detected using Dynabeads^®^ (Invitrogen). After transfection cells were incubated at 37°C (5% CO_2_) for 6 h before plating them on small dry‐heat sterilized glass coverslips. Electrophysiological experiments were conducted after at least 24 h of further incubation at 37°C (5% CO_2_). Throughout the Results section, hERG refers to hERG1a, except for data in Figure 6, which were obtained from coexpressed hERG1a/1b.

### Electrophysiological recording

Coverslips with plated cells were placed in a recording chamber mounted on an inverted microscope (Nikon Diaphot, Kingston upon Thames, UK). The chamber temperature was kept at 37°C and cells were continuously superfused with a standard Tyrode's solution containing (in mmol/L): 140 NaCl, 4 KCl, 2.5 CaCl_2_, 1 MgCl_2_, 10 glucose, 5 HEPES (titrated to pH 7.4 with NaOH) (Zhang et al. 2011; El Harchi et al. 2012; Du et al. 2014). Patch‐pipettes (Schott #8250 glass; A‐M Systems Inc., Sequim, WA) were pulled (Narishige, PP 830, Tokyo, Japan) and polished (Narishige, MF 83) to a final resistance between 2 and 4 MΩ. Patch‐pipettes were dialyzed with an intracellular solution containing (in mmol/L): 130 KCl, 1 MgCl_2_, 5 EGTA, 5 MgATP, 10 HEPES (titrated to pH 7.2 with KOH) (Zhang et al. 2011; El Harchi et al. 2012; Du et al. 2014). *I*_hERG_ was recorded using an Axopatch 1D or 200B amplifier (Axon Instruments, now Molecular Devices) and a CV‐4/100 or CV203BU head‐stage. Voltage‐clamp commands were generated with Clampex 8 or Clampex 9.2 (Axon Instruments, now Molecular Devices). Pipette series resistance was compensated between 70% and 80%. Data were acquired through a Digidata 1200B or a Digidata 1320A (Axon Instruments, now Molecular Devices). Data digitization rates were 10–25 kHz during all protocols and an appropriate bandwidth of 2–10 kHz was set on the amplifier.

### Potassium solutions

The standard Tyrode's solution described earlier was modified to simulate hypo‐ and hyperkalemic conditions. Low [K^+^]_e_ solution was made by lowering the KCl in the Tyrode's solution from 4 to 1 mmol/L, while the raised [K^+^]_e_ solution contained 10 mmol/L KCl. In both cases, the NaCl concentration was adjusted accordingly to maintain the same total external [K^+^] + [Na^+^]: when [K^+^]_e_ was reduced to 1 mmol/L, [Na^+^]_e_ was increased by 3 mmol/L and when [K^+^]_e_ was increased to 10 mmol/L, [Na^+^]_e_ was reduced by 6 mmol/L. All the solutions were warmed at 37°C and superfused over the cells using a homemade, multibarreled perfusion system that allowed rapid exchange of extracellular solutions (Levi et al. 1996).

### Data analysis

All data analysis was performed using Clampfit 10.3 and 10.2 (Axon Instruments, now Molecular Devices), Prism v4.03 and Excel 2003 and 2007. All data are presented as the mean ± SEM.

The effect of different external potassium concentrations on I_hERG_ “pulse” and “tail” currents was determined using the equation: 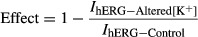
where *I*_hERG‐Altered[K_^+^_]_ and *I*_hERG‐Control_ represent “pulse” or “tail” currents in altered (hypo or hyperkalemia) and normal external potassium concentration. In both altered potassium conditions, a steady‐state was reached within ≈2 min and therefore no run‐down correction was needed.

The voltage dependence of inactivation was assessed using a three‐step protocol (Fig. 2A, inset) and by fitting the normalized peak currents with the equation: 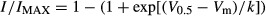
where *I* is amplitude of the peak current elicited by the third depolarizing step of the protocol after a brief 2 msec conditioning step (*V*_m_) that relieves the inactivation caused by the first depolarizing step. *I*_MAX_ is the maximal current amplitude during the third pulse observed during the protocol, and *V*_0.5_ and *k* are the half‐maximal inactivation voltage and the slope factor for the fit to the plotted relation.

To calculate the time constant of inactivation the transient current elicited by the third step of the three‐step protocol after a 2 msec step to −120 mV was fitted with a mono‐exponential equation: 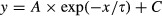
where *y* is the current amplitude at time *x*,* τ* is the time constant for the decay of the transient current, *A* represent the total fitted current, and *C* is the residual unfitted current component after the decline of the transient current.

**Figure 1.**
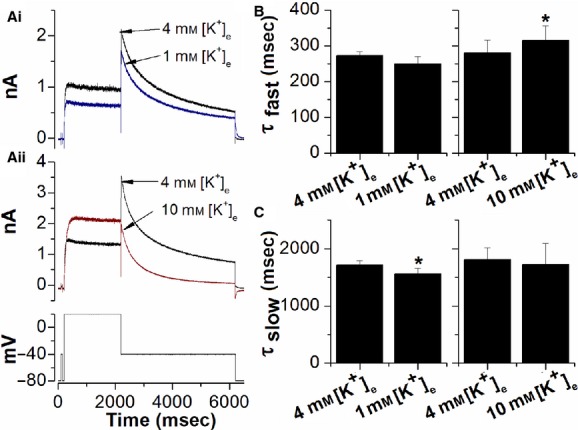
Effect of changing [K^+^]_e_ on *I*_hERG_. (A) Representative *I*_hERG_ traces showing the effect of lowering [K^+^]_e_ to 1 mmol/L (Ai) and raising [K^+^]_e_ to 10 mmol/L [K^+^]_e_ (Aii) on WT *I*_hERG_ elicited by the voltage protocol shown in the lower panel of Aii (successive applications of the protocol were at 12 sec intervals). (B) Effects of changing extracellular K^+^ concentration on the fast time constant of *I*_hERG_ deactivation on repolarization to −40 mV. 1 mmol/L (left) and 10 mmol/L (right) [K^+^]_e_ were studied separately, so pairwise comparisons were made between groups of cells where [K^+^]_e_ was switched from 4 to 1 mmol/L (31 cells) and from 4 to 10 mmol/L (15 cells). (C) Effects of changing extracellular K^+^ concentration on the slow time constant of deactivation. Groups were as for “B”. In both B and C “*” denotes statistical significance of *P* < 0.05 (paired *t*‐test).

Similarly, the time constants of deactivation were assessed by fitting the decaying tail current elicited by a standard *I*_hERG_ protocol (Fig. 1) with a double‐exponential function: 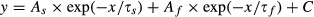
where *y* is the current amplitude at time *x*,* τ*_*s*_ and *τ*_*f*_ are the slow and the fast time constants of the slow and fast components of tail current deactivation. *A*_*s*_ and *A*_*f*_ represent the total current fitted by the fast and the slow components and *C* is the residual unfitted current.

Statistical analysis was performed using a paired, unpaired *t*‐test or a two‐way ANOVA (analysis of variance) with Bonferroni post‐test, as appropriate. *P* values less than 0.05 were considered to be statistically significant.

## Results

### Effects of altering [K^+^]_e_ on *I*_hERG_ elicited by a standard square pulse protocol

In initial experiments, the effects of reducing [K^+^]_e_ from 4 to 1 mmol/L and elevating it from 4 to 10 mmol/L were assessed using a conventional voltage protocol, employed in a number of prior studies of *I*_hERG_ from our laboratory (e.g., Du et al. 2010, 2011, 2013), in which membrane potential was stepped from −80 to +20 mV for 2 sec and then repolarized to −40 mV, in order to observe *I*_hERG_ tails (see lower panel of Fig. 1Aii). A brief (50 msec) depolarization was incorporated before the protocol in order to monitor instantaneous leak current at −40 mV, which was used as a reference level for measuring tail current amplitude (Du et al. 2010, 2011, 2013). Figure 1Ai shows *I*_hERG_ elicited in 4 mmol/L [K^+^]_e_ and, in the same cell, 2 min after switching to 1 mmol/L [K^+^]_e_ superfusate. This intervention resulted in reduced *I*_hERG_ during both the +20 mV step and during the −40 mV repolarization step. In 31 cells, the mean reduction in *I*_hERG_ during the +20 mV step was 31.5 ± 1.0%, while the *I*_hERG_ tail on repolarization was reduced by 21.8 ± 1.6%. Figure 1Aii shows data from a separate experiment in which [K^+^]_e_ was switched from 4 to 10 mmol/L. This resulted in an increase in *I*_hERG_ during the +20 mV step and a reduction in *I*_hERG_ tail current. In 15 cells, the mean increase in *I*_hERG_ during the +20 mV depolarization was 33.7±7.5% of the step *I*_hERG_, while the *I*_hERG_ tail on repolarization was decreased by 38.9 ± 3.8%. This differential effect of raising [K^+^]_e_ on pulse and tail currents is consistent with prior data on *I*_Kr_/*I*_hERG_ (Sanguinetti et al. 1995; Yang et al. 1997). In order to determine whether or not altering [K^+^]_e_ affected *I*_hERG_ deactivation time‐course, the *I*_hERG_ tails in each condition were fitted with equation 4 (Methods) to derive fast and slow deactivation time constants (*τ*_fast_ and *τ*_slow_, respectively). Reducing [K^+^]_e_ from 4 to 1 mmol/L did not significantly alter *τ*_fast_ of deactivation and produced only a small (~10%) decrease in *τ*_slow_ (Fig. 1B; *P* < 0.05 vs. 4 mmol/L). Raising [K^+^]_e_ from 4 to 10 mmol/L did not significantly alter *τ*_slow_ of deactivation and produced only a small (~4%) increase in *τ*_fast_ (Fig. 1C; *P* < 0.05 vs. 4 mmol/L).

### Effects of altering [K^+^]_e_ on *I*_hERG_ inactivation

**Figure 2.**
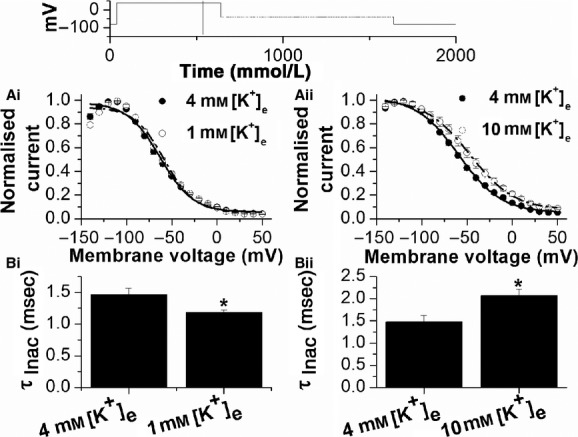
Effects of changing [K^+^]_e_ on *I*_hERG_ inactivation. (A) Effects of different extracellular K^+^ concentration (Ai, 1 mmol/L [K^+^]_e_; Aii 10 mmol/L [K^+^]_e_) on the voltage dependence of *I*_hERG_ availability. Protocol is shown above as an inset. Normalized current values were obtained as described in the Results section, plotted against voltage and fitted with equation 2 (Methods). For Ai control V_0.5_ and *k* values in 4 mmol/L [K^+^]_e_ were −60.3 ± 1.3 mV and 21.7 ± 0.4 mV, while in 1 mmol/L [K^+^]_e_, V_0.5_ and *k* values were −61.1 ± 1.6 mV and 21.5 ± 0.6 (*P* > 0.5; *n* = 8). For Aii control V_0.5_ and *k* values in 4 mmol/L [K^+^]_e_ were −53.4 ± 2.0 mV and 25.0 ± 1.2 mV, while in 10 mmol/L [K^+^]_e_, V_0.5_ and *k* values were −43.6 ± 4.6 mV and 27.4 ± 0.9 (*P* < 0.05; *n* = 6). (B) Bar charts showing the effect of changing extracellular K^+^ concentration on the time constant of *I*_hERG_ inactivation at +40 mV (*τ*_inac_), following a brief (2 msec) hyperpolarizing step to −120 mV after an initial depolarization to +40 mV. *τ*_inac_ values were obtained by fitting the current at +40 mV following the brief hyperpolarization to −120 with equation 3 (Methods). Bi shows data for a reduction from 4 mmol/L to 1 mmol/L [K^+^]_e_ (*n* = 8), while Bii shows data for an increase from 4 to 10 mmol/L [K^+^]_e_ (*n* = 6). *Statistical significance of *P* < 0.05 (paired *t*‐test).

The voltage dependence of *I*_hERG_ availability (inactivation) was determined using the protocol shown as an inset above Figure 2A, which has been used in prior *I*_hERG_ investigations from our laboratory (Du et al. 2010, 2013). A 500 msec conditioning pulse from −80 to +40 mV to activate and inactivate *I*_hERG_ was followed by a brief (2 msec) repolarizing step to potentials between +50 and −140 mV, to relieve inactivation to varying extents, followed by a second depolarization to +40 mV. Current amplitude during this second +40 mV depolarization reflected the extent to which inactivation was relieved during the preceding 2 msec step. Current amplitudes were normalized to maximal current during the third step, corrected for *I*_hERG_ deactivation and plotted against repolarization step value, as described previously (McPate et al. 2005; Du et al. 2010, 2013). Figure 2Ai and 2Aii show resulting data plots in 4 mmol/L versus 1 mmol/L (Fig. 2Ai) and 4 mmol/L versus 10 mmol/L (Fig. 2Aii) [K^+^]_e_ (*n* = 8 and 6, respectively). The data were fitted with equation 2 to derive V_0.5_ and *k* values for voltage‐dependent inactivation of *I*_hERG_. Initial fits to the data yielded a ~2.4 mV positive shift in inactivation V_0.5_ on switching from 4 to 1 mmol/L [K^+^]_e_ and a ~13.0 mV positive shift in inactivation V_0.5_ on switching from 4 to 10 mmol/L [K^+^]_e_. However, time‐matched control measurements (over 6 min, in 4 mmol/L [K^+^]_e_) showed a modest ~3.1 mV (*n* = 5) positive shift in inactivation at time points correlating to those used to evaluate effects of 1 and 10 mmol/L [K^+^] and therefore derived V_0.5_ and *k* values were corrected accordingly. In paired experiments, V_0.5_ and *k* values in 4 mmol/L [K^+^]_e_ were −60.3 ± 1.3 mV and 21.7 ± 0.4 mV, while in 1 mmol/L [K^+^]_e_, V_0.5_ and *k* values were −61.1 ± 1.6 mV and 21.5 ± 0.6 (*P* > 0.5; *n* = 8). For cells used to evaluate the effect of 10 mmol/L [K^+^]_e,_ control V_0.5_ and *k* values in 4 mmol/L [K^+^]_e_ were −53.4 ± 2.0 mV and 25.0 ± 1.2 mV, while in 10 mmol/L [K^+^]_e_, V_0.5_ and *k* values were −43.6 ± 4.6 mV and 27.4 ± 0.9 (*P* < 0.05; *n* = 6). Thus, under our conditions, reducing [K^+^]_e_ from 4 to 1 mmol/L did not significantly alter the voltage dependence of *I*_hERG_ inactivation, while increasing [K^+^]_e_ from 4 to 10 mmol/L produced a ~+10 mV positive shift in inactivation V_0.5_. Figure 2Bi and Bii show time‐constant values (*τ*_inact_) for the development of inactivation at +40 mV, following relief of inactivation at −120 mV (Du et al. 2010, 2013). In 1 mmol/L [K^+^]_e_ the time‐course of inactivation was accelerated compared to in 4 mmol/L [K^+^]_e_, while in 10 mmol/L [K^+^]_e_ it was slowed (Fig. 2B). The rate of *I*_hERG_ recovery from inactivation was not significantly altered by [K^+^]_e_ (data not shown).

### Effects of lowering and increasing [K^+^]_e_ on S624A hERG

**Figure 3.**
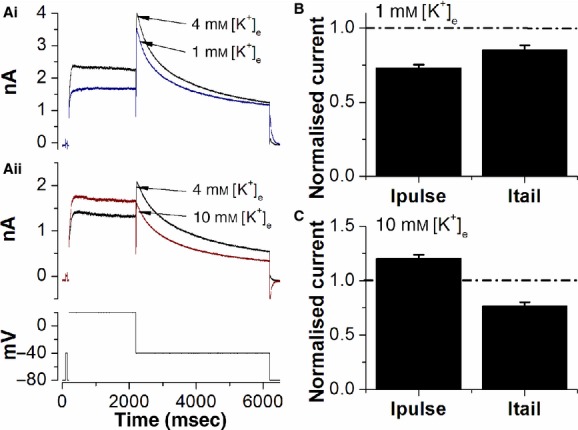
Effect of changing [K^+^]_e_ on S624A *I*_hERG_. (A) Representative *I*_hERG_ traces showing the effect of lowering [K^+^]_e_ to 1 mmol/L (Ai) and raising [K^+^]_e_ to 10 mmol/L [K^+^]_e_ (Aii) on S624A I_hERG_ elicited by the voltage protocol shown in the lower panel of Aii (successive applications of the protocol were at 12‐sec intervals). (B) Bar charts showing the effect of 1 mmol/L [K^+^]_e_ on I_hERG_ amplitude at the end of the 2 sec depolarization step to +20 mV (*I*_pulse_) and upon repolarization to −40 mV (*I*_tail_). For each experiment and each condition *I*_pulse_ and *I*_tail_ was normalized to the control value in 4 mmol/L [K^+^]_e_. Dashed‐dotted line is drawn at control level of 1 (*n* = 7). (C) Bar charts showing the effect of 10 mmol/L [K^+^]_e_ on *I*_hERG_ amplitude at the end of the 2 sec depolarization step to +20 mV (*I*_pulse_) and upon repolarization to −40 mV (*I*_tail_). For each experiment and each condition *I*_pulse_ and *I*_tail_ was normalized to the control value in 4 mmol/L [K^+^]_e_. Dashed‐dotted line is drawn at control level of 1 (*n* = 5).

Chronic exposure to low [K^+^]_e_ has been proposed to decrease surface membrane I_Kr_/hERG through induction of a novel nonconducting state and promotion of channel internalization/degradation (Guo et al. 2009; Massaeli et al. 2010). Removal of external K^+^ (0 mmol/L [K^+^]_e_) was suggested to be able to induce the nonconducting state for wild‐type (WT) hERG within minutes, but not to be able to do so for channels comprising the S624A hERG pore mutant (Massaeli et al. 2010). In order to ascertain whether such a mechanism might contribute to the [K^+^]_e_ induced changes in WT *I*_hERG_ amplitude shown in Figure 1, we performed similar experiments on S624A *I*_hERG_ to those shown in Figure 1. Figure 3Ai and Aii demonstrate that decreasing and increasing [K^+^]_e_ produced qualitatively similar effects on S624A *I*_hERG_ to those seen for WT *I*_hERG_ under our conditions. Mean data for 1 mmol/L [K^+^]_e_ are shown in Figure 3B. Lowering [K^+^]_e_ from 4 to 1 mmol/L induced reductions of 26.8 ± 2.1% and 14.5 ± 2.9% of step and tail *I*_hERG_, respectively (*n* = 7 cells; *P* > 0.05 vs. WT hERG for both). The similar responses of WT and S624A *I*_hERG_ to acute exposure to 1 mmol/L [K^+^]_e_ under our conditions indicate that WT current amplitude reductions with lowered [K^+^]_e_ in our experiments are unlikely to be attributable to induction of the (S624A‐hERG sensitive) nonconducting state reported by Massaeli et al. (2010). Mean data for 10 mmol/L [K^+^]_e_ on S624A *I*_hERG_ are shown in Figure 3C. Increasing [K^+^]_e_ from 4 to 10 mmol/L resulted in an increase of 20.3 ± 3.6% of the step *I*_hERG_ (*P* > 0.05 vs. WT; *n* = 5 cells) and a decrease of 23.1 ± 3.4% of the *I*_hERG_ tail (*P* < 0.01 vs. WT; *n* = 5 cells for both). The lack of significant difference between the response of WT and S624A *I*_hERG_ during the +20 mV test command is suggestive that any difference in tail current response likely resulted from differences in gating of the two channels rather than in surface expression (though any such differences were beyond the intended scope of this study and so were not pursued).

### Effects of lowering and increasing [K^+^]_e_ on the response of hERG to premature stimulation

**Figure 4.**
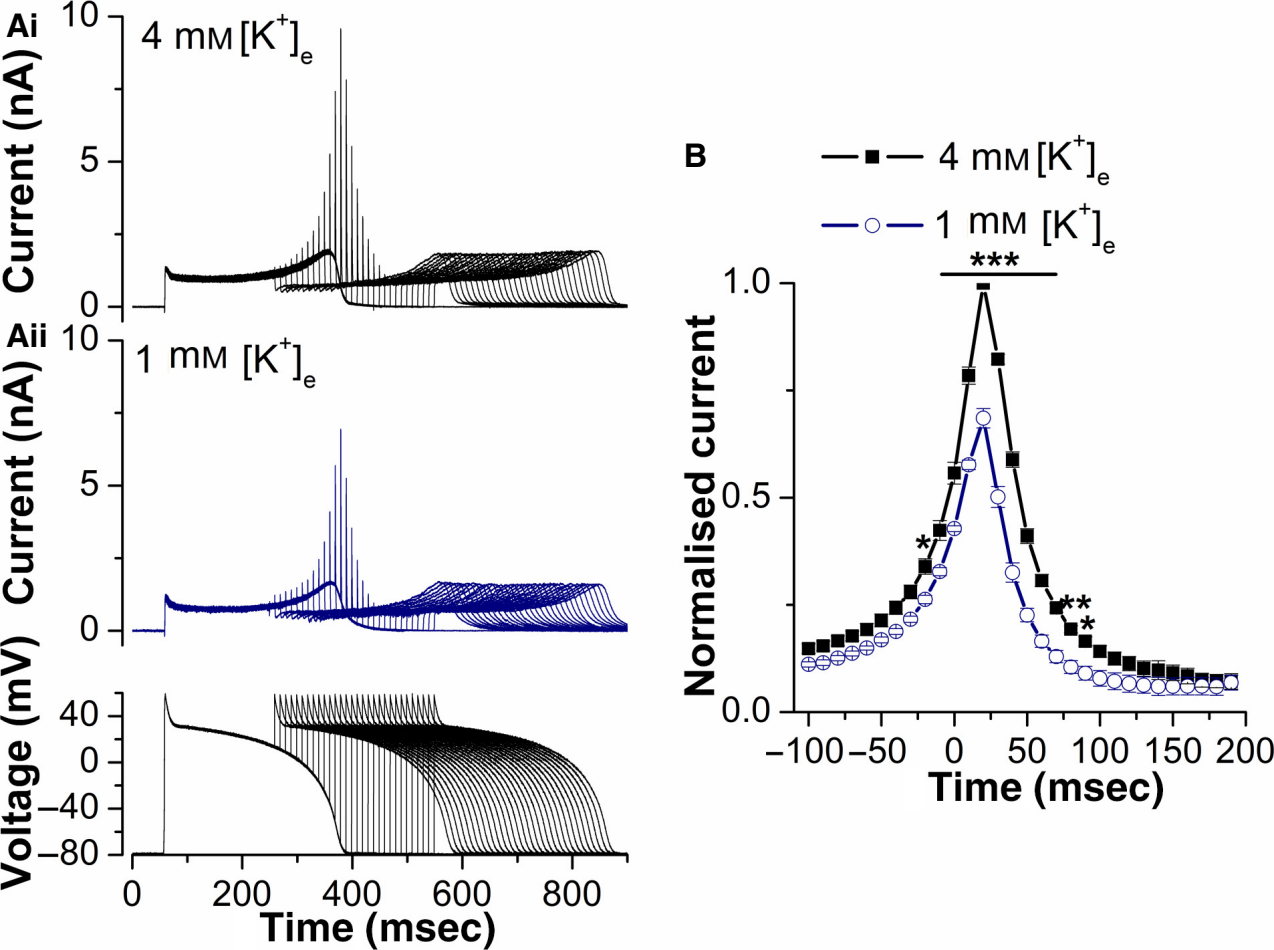
Effect of reducing [K^+^]_e_ on the response of *I*_hERG_ to premature stimulation. (A) Example traces of hERG (1a) current recorded at 4 mmol/L [K^+^]_e_ (Ai) and 1 mmol/L [K^+^]_e_ (Aii) elicited by the protocol that comprised paired ventricular AP command waveforms shown in the lower panel of Aii. The second AP was introduced at times corresponding to APD_90_ − 100 msec up to APD_90_ + 190 msec of the first AP. (B) Plots of the normalized amplitude of outward current transients during the paired ventricular AP command waveforms against the interpulse interval at 4 and 1 mmol/L [K^+^]_e_. The *I*_hERG_ transients at each time‐point were normalized to the peak *I*_hERG_ transient amplitude observed in control (4 mmol/L [K^+^]_e_) in each individual cell. Reducing [K^+^]_e_ from 4 to 1 mmol/L produced a decrease of 31.5 ± 2.3% (*n* = 7 cells) in maximal *I*_hERG_ transient amplitude. Statistical significance of *P < 0.05; **P < 0.01; ***P < 0.001 (two‐way ANOVA, with Bonferroni post hoc pairwise comparison).

In order to ascertain the effect of [K^+^]_e_ on the *I*_hERG_ response to premature stimulation, a pulse protocol was used that comprised paired AP waveforms, in which an initial and second AP command were separated by varying intervals following the application of the first AP, with the second AP applied both before and following completion of initial AP repolarization (cf. McPate et al. 2009; Du et al. 2010, 2013). Figure 4A shows representative *I*_hERG_ traces elicited by this protocol in 4 mmol/L [K^+^]_e_ and following application of 1 mmol/L [K^+^]_e_ (Fig. 4Ai and Aii, respectively, with the protocol shown as the lower panel of Fig. 4Aii). Under both conditions the second AP command elicited rapid transient currents, before a sustained component similar to that elicited by the first AP. The magnitude of *I*_hERG_ during the first AP was reduced following application of 1 mmol/L [K^+^]_e_: the current at the start of the plateau immediately after phase 1 repolarization was reduced by 15.2 ± 4.3%, while the maximal current during repolarization was reduced by 13.6 ± 1.7% (*n* = 7 for both). The overall pattern of rapid *I*_hERG_ transients was similar between 4 and 1 mmol/L [K^+^]_e_ (with maximal *I*_hERG_ transient amplitude at ~20 msec following 90% repolarization [APD_90_] of the first AP; Lu et al. 2001; McPate et al. 2009; Du et al. 2010, 2013), but the amplitude of the transients was reduced at the lower [K^+^]_e_ (Fig. 4Ai, Aii, and B). Figure 4B shows mean data from seven such experiments. This reduction was statistically significant for time‐points between 20 msec preceding APD_90_ of the first AP and 90 msec after APD_90_. Thus, over this time‐frame, lowering [K^+^]_e_ reduced the response of *I*_hERG_ to premature stimuli, with the maximal response reduced by 31.5 ± 2.3% (*n* = 7).

**Figure 5.**
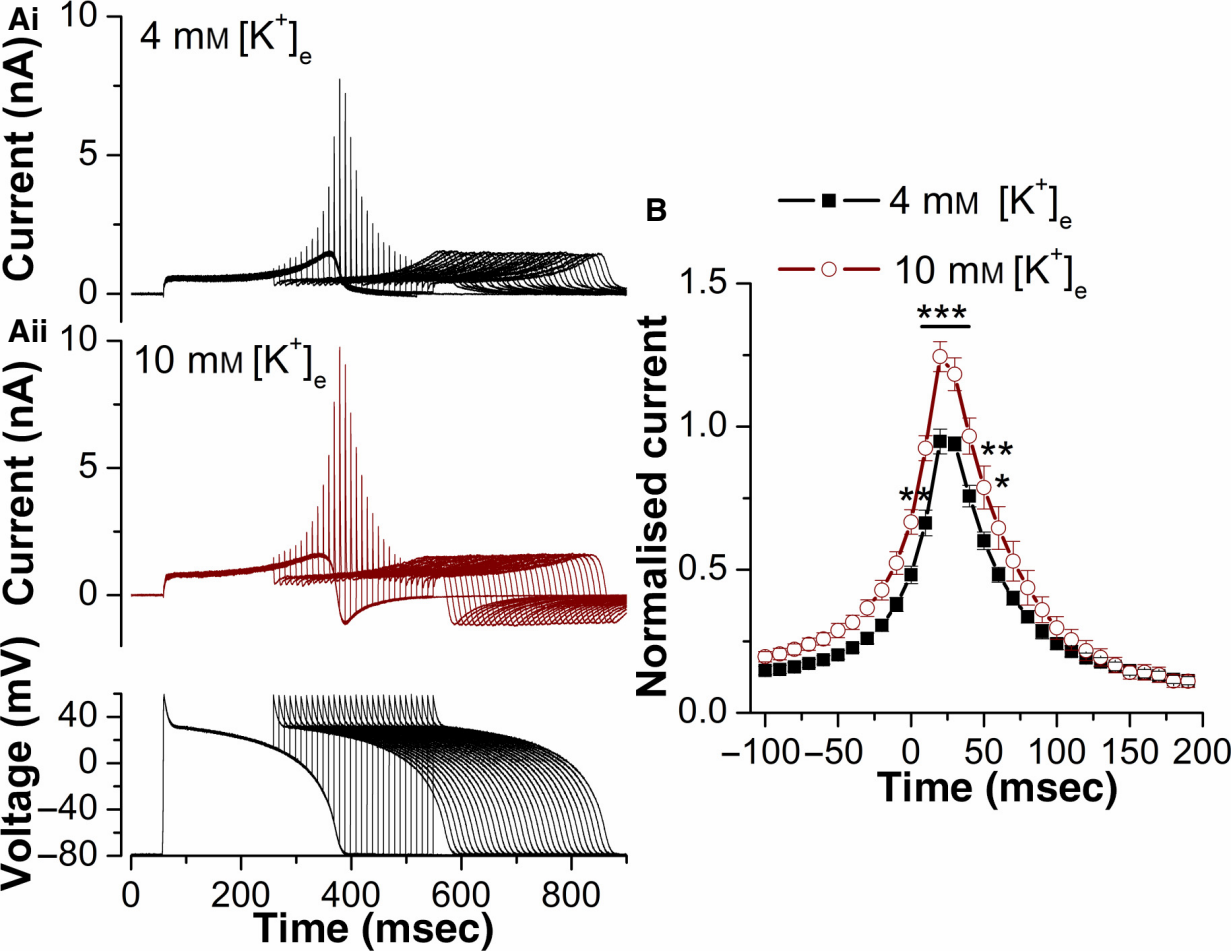
Effect of raising [K^+^]_e_ on the response of *I*_hERG_ to premature stimulation. (A) Example traces of hERG (1a) current recorded at 4 mmol/L [K^+^]_e_ (Ai) and 10 mmol/L [K^+^]_e_ (Aii) elicited by the protocol that comprised paired ventricular AP command waveforms shown in the lower panel of Aii. The second AP was introduced at times corresponding to APD_90_ − 100 msec up to APD_90_ + 190 msec of the first AP. (B) Plots of the normalized amplitude of outward current transients during the paired ventricular AP command waveforms against the interpulse interval at 4 mmol/L and 10 mmol/L [K^+^]_e_. The *I*_hERG_ transients at each time‐point were normalized to the peak *I*_hERG_ transient amplitude observed in control (4 mmol/L [K^+^]_e_) in each individual cell. Increasing [K^+^]_e_ from 4 to 10 mmol/L produced an increase of 24.9 ± 5.6% (*n* = 7 cells) in maximal *I*_hERG_ transient amplitude. Statistical significance of *P < 0.05; **P < 0.01; ***P < 0.001 (two‐way ANOVA, with Bonferroni post hoc pairwise comparison).

Figure 5 shows the response of *I*_hERG_ to the same premature stimulation protocol, when [K^+^]_e_ was raised from 4 to 10 mmol/L. The response of *I*_hERG_ during the initial AP was mixed: current immediately following the phase 1 repolarization was increased (by 26.8±8.3% *n* = 7 cells), while the maximal current during repolarization was insignificantly reduced (by 0.6 ± 3.9%; *n* = 7). The differential effects of raised [K^+^]_e_ on *I*_hERG_ at positive voltages early in the AP and the peak current later in repolarization (which occurred at ~−30 to −40 mV) are analogous to those seen with conventional voltage‐clamp in Figures 1Aii and 3Aii. Deactivating current following complete AP repolarization was inward in 10 mmol/L [K^+^]_e_ due to the positively shifted equilibrium potential for K^+^ compared to the −80 mV holding potential. The response to premature stimuli was augmented, however (Fig. 5Ai, Aii and B). Maximal *I*_hERG_ transient amplitude was increased by 24.9 ± 5.6% (*n* = 7 cells) and statistically significant increases were seen between APD_90_ and 60 msec following APD_90_ of the first AP.

**Figure 6.**
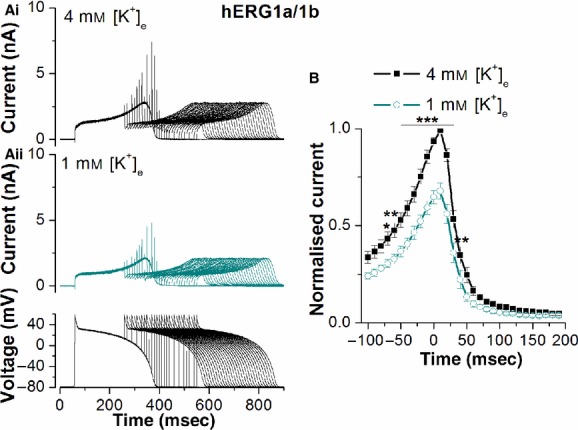
Effect of reducing [K^+^]_e_ on the response of *I*_hERG_ carried by hERG1a/1b. (A) Example traces of hERG 1a/1b current recorded at 4 mmol/L [K^+^]_e_ (Ai) and 1 mmol/L [K^+^]_e_ (Aii) elicited by the protocol that comprised paired ventricular AP command waveforms shown in the lower panel of Aii. The second AP was introduced at times corresponding to APD_90_ − 100 msec up to APD_90_ + 190 msec of the first AP. (B) Plots of the normalized amplitude of outward current transients during the paired ventricular AP command waveforms against the interpulse interval at 4 and 1 mmol/L [K^+^]_e_. The *I*_hERG_ transients at each time‐point were normalized to the peak *I*_hERG_ transient amplitude observed in control (4 mmol/L [K^+^]_e_) in each individual cell. Reducing [K^+^]_e_ from 4 to 1 mmol/L produced a decrease of 32.1 ± 4.1% (*n* = 8 cells) in maximal *I*_hERG_ transient amplitude. *Statistical significance of *P < 0.05; **P < 0.01; ***P < 0.001 (two‐way ANOVA with Bonferroni post hoc pairwise comparison).

Although most studies of recombinant hERG focus on the hERG1a isoform, there is some evidence that native I_Kr_ channels comprised hERG1a coassembled with the shorter hERG1b isoform (e.g., London et al. 1997; Jones et al. 2004; Sale et al. 2008). For completeness, therefore, in a final series of experiments we investigated whether the effects of reducing [K^+^]_e_ on the response to premature stimulation are preserved when hERG1a is coexpressed with hERG1b rather than alone. Figure 6 shows the results of these experiments. Similar to the situation for hERG1a (Fig. 4), *I*_hERG_ carried by hERG1a/1b was reduced when [K^+^]_e_ was switched from 4 to 1 mmol/L (Fig. 6Ai and ii). Immediately after phase 1, repolarization of the initial AP, *I*_hERG_ was reduced by 20.8 ± 5.7% (*n* = 8 cells; *P* > 0.05 vs. hERG1a), while maximal *I*_hERG_ during AP repolarization was reduced by 23.7 ± 4.8% (*n* = 8; *P* > 0.05 vs. hERG1a). As shown by the representative traces in Figure 6Ai and ii the rapid *I*_hERG_ transients induced by premature stimulation were reduced by exposure to 1 mmol/L [K^+^]_e_. Figure 6B shows mean data. In both 4 and 1 mmol/L [K^+^]_e_ the relationship descended more steeply following the maximal response that seen for hERG1a; this is attributable to the known more rapid deactivation kinetics for hERG1a/1b than hERG1a alone (London et al. 1997; Jones et al. 2004; Sale et al. 2008). Reducing [K^+^]_e_ from 4 to 1 mmol/L produced a decrease of 32.1 ± 4.1% in maximal *I*_hERG_ transient amplitude (*n* = 8 cells; *P* > 0.5 vs. response for hERG1a) and *I*_hERG_ transient amplitude was significantly smaller in 1 mmol/L than 4 mmol/L [K^+^]_e_ between 70 ms preceding APD_90_ and 40 ms following APD_90_ of the initial AP command. Consequently, lowering [K^+^]_e_ reduced the *I*_hERG_ response to premature stimulation both for hERG1a and for hERG1a/1b.

## Discussion

### Results in context

A paradoxical effect of altering [K^+^]_e_ on *I*_hERG_ amplitude was observed in early studies of *I*_Kr_ and hERG. Thus, acutely lowering [K^+^]_e_ from 4 to 0 mmol/L was reported to increase the amplitude of *slow* delayed rectifier current, *I*_Ks_, in guinea‐pig ventricular myocytes (consistent with the expectation from the altered driving force for K^+^ ions), while it reduced *I*_Kr_ from the same preparation (Sanguinetti and Jurkiewicz 1992). Subsequently, in one of the first studies of hERG, utilizing *Xenopus* oocyte expression, increasing superfusing [K^+^]_e_ from 2 to 10 mmol/L increased pulse current and decreased tail current, while in contrast exposure to 0 mmol/L [K^+^]_e_ reduced both pulse and tail currents (Sanguinetti et al. 1995). Qualitatively similar results were observed for *I*_Kr_ from AT‐1 cells over a [K^+^]_e_ range from 1 to 8 mmol/L (Yang and Roden 1996). The present results for WT *I*_hERG_ recorded at 37°C from a mammalian cell expression system (Fig. 1) are in qualitative agreement with the findings of these earlier studies.

In 1997, further work on *I*_Kr_ from AT‐1 cells showed that decreasing [K^+^]_e_ led to smaller inactivation time constant values, implicating hERG's rapid inactivation in the modulatory effect of [K^+^]_e_ (Yang et al. 1997). The same year Wang et al. (1997a) demonstrated that inactivation of *I*_hERG_ recorded from *Xenopus* oocytes was shifted by +30 mV and the inactivation time course was also slowed when [K^+^]_e_ was raised from 2 to 98 mmol/L; voltage‐dependent activation was unaffected. An independent study the following year, also using hERG expressed in *Xenopus* oocytes reported that raising [K^+^]_e_ from 2 to 20 mmol/L shifted inactivation V_0.5_ by +20 mV (Zou et al. 1998). Through the use of different alkali, cations, and TEA, Shimizu et al. (2003) located the inactivation‐impeding site toward the external face of the channel, in the selectivity filter close to the TEA‐binding site at the entrance to the filter . Clearly, therefore, there are substantial data in the literature supporting a modulatory effect of changes in [K^+^]_e_ on *I*_hERG_ inactivation, through K^+^ ions acting at a site toward the channel exterior. It is noteworthy, however, that in simulating the effects of [K^+^]_e_ on *I*_hERG_, Wang et al. (1997b) concluded that altered inactivation alone was insufficient to account for the effects of raised [K^+^]_e_ on macroscopic *I*_hERG_ and that a significant increase in total conductance is likely also to be involved. More recently, chronic changes in [K^+^]_e_ have been reported to lead to changes to cell surface expression of hERG and to the functional expression of native *I*_Kr_ channels (Guo et al. 2009; Massaeli et al. 2010). Thus, K^+^ removal (0 mmol/L [K^+^]_e_) was reported to drive WT hERG channels into a nonconducting state, followed by subsequent internalization and degradation (Massaeli et al. 2010). This sequence of events was supported by the effects of comparatively brief exposure to 0 mmol/L [K^+^]_e_, which was suggested to enable the nonconducting state, but without changes in channel expression evident with longer duration exposure (Massaeli et al. 2010). Mutations in the pore‐helix/selectivity filter, including the S624A mutation employed in the present study, were able to inhibit the response to chronic K^+^_e_ removal (Massaeli et al. 2010). The present results on the acute effects of [K^+^]_e_ modulation of *I*_hERG_ appear not to be attributable to such a mechanism: WT and S624A *I*_hERG_ responded similarly to one another (Figs 1 and 3) to reduction (to 1 mmol/L) or elevation (to 10 mmol/L) of [K^+^]_e_ from the control level of 4 mmol/L. We observed significant changes to inactivation time‐constant both on lowering and elevating [K^+^]_e_, while raising [K^+^]_e_ from 4 to 10 mmol/L also resulted in a positive shift in voltage‐dependent inactivation (Fig. 2), in qualitative agreement with previous studies (Wang et al. 1997a; Zou et al. 1998). The significant shift in voltage‐dependent inactivation in addition to accelerated inactivation time‐course is likely to account for the concomitant increase in pulse *I*_hERG_ and decrease in tail *I*_hERG_ seen with raised [K^+^]_e_ (Figs 1 and 3). However, a potential contribution of altered hERG channel conductance to the overall effect (as suggested by Wang et al. 1997a) cannot be ruled out, given that single hERG channel conductance is known to vary with [K^+^]_e_ (2 pS at 5 mmol/L and 10 pS at 100 mmol/L in Kiehn et al. 1996). Although *I*_hERG_ is known to be sensitive to [Na^+^]_e_ (Namaguchi et al. 2000; Mullins et al. 2002) and changes to [K^+^]_e_ in the present study were compensated by concomitant alterations to [Na^+^]_e_, the modulatory effects of [Na^+^]_e_ on *I*_hERG_ amplitude are most marked for [Na^+^]_e_ concentrations substantially below 100 mmol/L (Namaguchi et al. 2000; Mullins et al. 2002) and so are unlikely to contribute significantly to observed effects of altering [K^+^]_e_ in our experiments.

### The response to premature stimulation under AP clamp

The profile of WT *I*_hERG_ seen here in normal (4 mmol/L) [K^+^]_e_ both during imposed AP clamp commands and in response to premature AP stimuli (Figs 4 and 5) is comparable to that found in prior studies that have used similar paired AP clamp protocols, with maximal *I*_hERG_ transient amplitude occurring when premature stimuli were applied shortly after the point of 90% complete repolarization of the first AP (APD_90_) (Lu et al. 2001, 2003; McPate et al. 2009; Du et al. 2010). Application of premature stimuli between 100 msec before APD_90_ of the initial AP and 190 msec after APD_90_ was sufficient to reveal the normal biphasic relationship of *I*_hERG_ transient amplitude with time late in repolarization/early in diastole (Lu et al. 2001, 2003; McPate et al. 2009; Du et al. 2010). In our experiments, reduced [K^+^]_e_ decreased *I*_hERG_ both during the initial AP command and during the transient responses to the second AP command waveform. To our knowledge, our data constitute the first direct AP clamp demonstration of modification by [K^+^]_e_ of the *I*_hERG_ response to premature stimulation. We have shown previously a suppression of the *I*_hERG_ response to premature stimuli in the context of extracellular acidosis, an effect that was associated with marked acceleration of *I*_hERG_ deactivation (Du et al. 2010). However, in the case of low [K^+^]_e_, the fast component of deactivation was unaffected by reducing [K^+^]_e_ from 4 to 1 mmol/L (Fig. 1) and so the altered response to premature stimuli in late repolarization/early diastole is unlikely to be accounted for by changes to *I*_hERG_ deactivation. Rather, enhanced inactivation and reduced net conductance are likely to account for the reduced response to premature stimuli. It is significant that coexpressed hERG1a/1b showed a similar suppression of the *I*_hERG_ response to premature stimuli with low [K^+^]_e_ to that of hERG1a alone (Figs 4 and 6). Thus, whether native *I*_Kr_ results from heteromeric hERG1a and hERG1b (London et al. 1997; Jones et al. 2004; Sale et al. 2008) or from hERG1a alone, it is safe to conclude that the channel's protective role against premature depolarization at time‐points comparable to those studied here is likely to be significantly reduced in circumstances with reduced [K^+^]_e_.

The characteristic resurgent *I*_hERG_ tail during conventional voltage‐clamp results from rapid recovery of *I*_hERG_ from inactivation on membrane potential repolarization. Concomitant increases in *I*_hERG_ pulse current and decreases in tail current with raised [K^+^]_e_ (Figs 1 and 3; Sanguinetti et al. 1995; Yang and Roden 1996) are both consequences of attenuated inactivation. The effect of 10 mmol/L [K^+^]_e_ on *I*_hERG_ during the AP waveform seen here reflects *dynamic* changes in *I*_hERG_ gating during the AP, such that peak *I*_hERG_ during repolarization (which typically occurs between ~−30 and −40 mV; Hancox et al. 1998; McPate et al. 2005) was little changed, but *I*_hERG_ early during the AP was increased. Thus, an increased contribution of *I*_Kr_ to repolarization might be anticipated early during the ventricular AP under situations of hyperkalemia. Our data are also suggestive of an increased ability of hERG to resist premature depolarization for a short period early in diastole.

### Potential physiological significance

In the setting of experimental acute coronary occlusion or ischemia, [K^+^]_e_ accumulation to values exceeding 10 mmol/L has been reported (Hill and Gettes 1980; Weiss and Shine 1982). Consequently, our data with raised [K^+^]_e_ have relevance in terms of suggesting an altered role of *I*_Kr_ both early during the ventricular AP plateau and late in repolarization/early in diastole (as considered earlier). If pathological ischemia/K^+^ accumulation is localized, then the localized effect of raised [K^+^]_e_ on hERG/*I*_Kr_ could contribute to heterogeneity in repolarization and in tissue sensitivity to premature excitation. On the other hand, global hypokalemia is strongly associated with risk of arrhythmia and is known to exacerbate the risk of acquired (drug‐induced) LQTS and associated *Torsades de Pointes* (TdP) (Viskin 1999; Zeltser et al. 2003). In profound hypokalemia levels close to 1 mmol/L (1.2 mmol/L) have been reported (Garcia et al. 2008). Thus, while the reduction in [K^+^]_e_ from 4 to 1 mmol/L can fairly be considered to represent an extreme in terms of clinically relevant hypokalemia, our findings constitute a valuable proof‐of‐concept demonstration: acute hypokalemia not only reduces the contribution of *I*_hERG_/*I*_Kr_ to ventricular repolarization but can also impair the channel's protective role against premature excitation. In chronic hypokalemia, these acute effects can be expected to be synergistic with decreased surface expression of *I*_Kr_/hERG channels consequent to sustained low [K^+^]_e_ (Guo et al. 2009; Massaeli et al. 2010), to contribute to the overall effect. In the additional presence of a hERG/*I*_Kr_ blocking drug, these effects can be anticipated to combine with pharmacological suppression of *I*_hERG_ in augmenting the overall arrhythmic risk. Conversely, restoration of a normal [K^+^]_e_ in hypokalemic patients can be anticipated to restore both the role of hERG/I_Kr_ in normal ventricular repolarization and its protective role early in diastole. It is feasible that acute effects of raising [K^+^]_e_ on *I*_hERG_ may contribute to the beneficial actions of potassium supplementation therapy (raising serum potassium by ~1 mmol/L) in patients with hERG mutation‐linked congenital LQTS (Compton et al. 1996; Etheridge et al. 2003), although the effects of long‐term potassium supplementation in that setting are perhaps more likely to involve [K^+^]_e_ linked changes to cell surface channel expression (Guo et al. 2009; Massaeli et al. 2010).

## Conflict of Interest

None declared.
